# Neurobiology of Spirituality

**DOI:** 10.4103/0973-1229.33001

**Published:** 2008

**Authors:** E. Mohandas

**Affiliations:** * *Chief Consultant Psychiatrist, Elite Mission Hospital, Kerala, India*

**Keywords:** *Anxiety and Psychotic states*, *Depression*, *Functional Imaging*, *Meditation*, *Prefrontal Hypothesis*, *PSPL Deafferentation*, *Spirituality*

## Abstract

Spiritual practices have been proposed to have many beneficial effects as far as mental health is concerned. The exact neural basis of these effects is slowly coming to light and different imaging techniques have elucidated the neural basis of meditative practices. The evidence though preliminary and based on studies replete with methodological constraints, points toward the involvement of the prefrontal and parietal cortices. The available data on meditation focus on activated frontal attentional network. Neuroimaging studies have shown that meditation results in an activation of the prefrontal cortex, activation of the thalamus and the inhibitory thalamic reticular nucleus and a resultant functional deafferentation of the parietal lobe. The neurochemical change as a result of meditative practices involves all the major neurotransmitter systems. The neurotransmitter changes contribute to the amelioration of anxiety and depressive symptomatology and in part explain the psychotogenic property of meditation. This overview highlights the involvement of multiple neural structures, the neurophysiological and neurochemical alterations observed in meditative practices.

## Introduction

Spirituality is defined as that relating to or consisting of or having the nature of spirit. The nature of spirit is intangible or immaterial. The English word ‘spirit’ comes from the Latin ‘*spiritus*’ meaning breath. The spiritual realm deals with the perceived eternal realities regarding man's ultimate nature, in contrast to what is temporal or worldly. Spirituality involves as its central tenet a connection to something greater than oneself, which includes an emotional experience of religious awe and reverence. Spirituality is therefore an individual's experience of and relationship with a fundamental, nonmaterial aspect of the universe that may be referred to in many ways – God, Higher Power, the Force, Mystery and the Transcendent and forms the way by which an individual finds meaning and relates to life, the universe and everything.

Religious experience and religion forms only a part of a person's spiritual quest. Religion is an organized belief system promulgated and sustained by a human institution, ethnic group, tribe or culture and involves definite rules of behaviour, practices and rituals. The English word religion comes from the Latin ‘*religio*’ meaning reverence, though a deeper study reveals it to be a combination of two words, ‘*Re*’ meaning return and ‘*Ligare*’ meaning ‘to bind’. Though closely related, religions probably originated as a way of meeting humanity's possible innate need for spirituality.

Spirituality and religion are not interchangeable or always linked. Therefore a person may have religion without spirituality or spirituality without religion. It is noted that spirituality can have both positive and negative effects on physical health, mental health and coping. The studies on how religion and spirituality affect the brain, though inconclusive, still give vital pointers to the neural mechanisms of such practices. Some of these are reviewed in this article.

## Spirituality and Mental Health

The role of spirituality as a resource for finding meaning and hope in suffering has also been identified as a key component in the process of psychological recovery. Most studies have shown that religious involvement and spirituality are associated with better health outcomes, including greater longevity, coping skills and health-related quality of life (even during terminal illness) and less anxiety, depression and suicide. Several studies have shown that addressing the spiritual needs of the patient may enhance recovery from illness (Mueller, *et al.*, 2001). A recent meta-analysis, which summarized the results of 147 independent investigations involving a total of 98,975 subjects on the association between religiousness and depressive symptoms, found that religiousness is modestly but robustly associated with lower level of depressive symptoms. It was also noted that two specific measures of religiousness, namely extrinsic religious orientation and negative religious coping had a positive association with high frequency of depressive symptoms, while intrinsic religious orientation was associated with low levels of depression (Moreira-Almeida, *et al.*, 2006). A study of the impact of religion and spirituality in schizophrenics showed that religion was used as a positive way of coping by 71% of patients and as a negative way of coping by 14%. The study found that religion and spirituality lessened (54%) or increased (10%), psychotic symptoms and may reduce (33%) or increase (10%), the risk of suicide attempts. It may also reduce (14%) or increase (3%), substance use and foster adherence to (16%) or be in opposition to (15%), psychiatric treatment (Mohr, *et al.*, 2006).

Of the 120 identified studies published prior to 2000 investigating religiousness and alcohol/drug use/abuse, most of them found a clear inverse correlation between these variables. A study conducted on students in a large metropolitan area showed that religious factors were strongly associated with lower drug abuse, even after controlling for the relevant socio-demographic and educational variables (Moreira-Almeida, *et al.*, 2006). The role of spirituality in addiction was also reflected upon in a study, which found that smoking and frequent binge drinking were negatively correlated with spirituality scores (Leigh, *et al.*, 2005).

A review of the 68 studies identified through 2000 found lower rates of suicide or more objections to suicide, among the more religious subjects. It was also noted that the level of religiousness was inversely associated with the acceptance of euthanasia and physician-assisted suicide in the general population in Britain, among the elderly in the US and cancer patients in a palliative care service in the US (Moreira-Almeida, *et al.*, 2006). A meta-analysis focusing on the usefulness of meditation therapy for anxiety disorders showed that transcendental meditation showed a reduction in anxiety symptoms and electromyography score comparable with electromyography-biofeedback and relaxation therapy (Krisanaprakornkit, *et al.*, 2006). A study also found that the relationships between religion/spirituality and mental health were generally stronger or more unique, for males and older adolescents than for females and younger adolescents (Wong, *et al.*, 2006). The evidence thus far points to a clear association, both positive and negative, between spirituality/religion and mental health.

## Neurobiological Explorations into Spirituality

There is a paucity of evidence regarding the neural correlates of spiritual practices and most studies that have explored spirituality have concentrated on meditative practices. Most of the studies examining them used functional imaging as the investigation tool, to delineate the neural mechanisms involved in these practices. They include the Positron Emission Tomography (PET) studies on Yoga, Tantric Yoga and Yoga Nidra; the Magnetic Resonance Imaging (MRI) study on Kundalini Yoga; and the Single Photon Emission Computerized Tomography (SPECT) study on Tibetan meditation. The studies point to prefrontal activation, transient hypofrontality, increased frontal lobe and decreased parietal lobe activity and also to a deafferentation of the posterior superior parietal lobule (PSPL) in spirituality [Table 1] (Newberg and Iversen 2003; Muramoto, 2003; Azari *et al.*, 2001).

**Table 1:** Neuroimaging Studies on Meditation (Adapted from Cahn and Polich, 2006)

Study	Method and sample	Findings

Herzog, *et al.*, 1990	PET, Yoga meditation (N = 8)	Increased fronto-parietal and fronto-occipital activation ratios, Slight decrease for posterior anterior ratios
Jevning, *et al.*, 1996	Rheoencephalography, Transcendental Meditation (TM) (N = 34)	Increased frontal and occipital blood flow
Lou, *et al.*, 1999	PET, Yoga Nidra (N = 9)	Increased CBF anterior parietal, fusiform gyrus, occipital cortex, Decreased in DLPFC, OFC, ACC, Lt. temporal, Lt. IPL, Striatal and Thalamic regions. B/L Hippocampal activation
Lazar, *et al.*, 2000	fMRI, Kundalini Yoga (N = 5)	Increased DLPFC, ACC, Parietal, Hc, Temporal, Striatal, Hypothalamic activity, 20% decrease in global blood flow
Khushu, *et al.*, 2000	fMRI, Raja Yoga (N = 11)	Increased PFC activity, Decreased activity in none
Baerensten, *et al.*, 2001	fMRI, Onset of meditation (N = 5)	Increased activation Lt. Frontal, Paracentral, Inferior Parietal, Lateral temporal, ACC, Hc, Activation also in Rt. Temporal Lobe, Sup. Parts of Rt. Gyrus paracentralis, Decreased activation occipital especially right, Decreased activation Post. Cingulate, Rt. Central Cortex
Newberg, *et al.*, 2001	SPECT, Tibetan meditation (N = 8)	Increased Cingulate, DLPFC, Inferior and OFC, Thalamus, Decreased PSPL, Increases in Lt. DLPFC correlated with decrease in Lt. PSPL
Azari, *et al.*, 2001	PET, Psalm 23 recitation (N = 12)	Activation of R-DLPF, DMFC and Rt. Precuneus, Religious experience a cognitive process mediated by circuit involving DLPF, DMFC and Medial parietal cortex
Kjaer, *et al.*, 2002	PET 11C-raclopride binding, Yoga Nidra (N = 5)	Decrease raclopride binding in ventral striatum, indicating increase dopamine binding
Newberg, *et al.*, 2003	SPECT, Franciscan Prayer (N = 3)	Increased blood flow in PFC, inferior parietal lobes, inferior frontal lobe. Inverse correlation between blood flow, changes in PFC and in the ipsilateral SPL.
Lazar, *et al.*, 2003	fMRI, Mindfulness vs. Kundalini (N = 33)	Different distribution of activated networks in the 2 groups. Both showed increased Cingu-late activation. Rt.TL activation only in mindfulness
Ritskes, *et al.*, 2003	fMRI, Zen (N = 11)	Increased activity in DLPFC (R > L), basal ganglia. Decreased in right anterior superior occipital gyrus, ACC
Lazar, *et al.*, 2005	MRI, Buddhist Insight Meditation (N = 20)	Brain regions associated with attention, interoception and sensory processing was thicker in meditators than matched controls. PFC and Rt. Ant. Insula. Experience dependent cortical plasticity
Kakigi, *et al.*, 2005	fMRI, Noxious LASER stimulation of a Yoga master who claims to feel no pain during meditation	Decreased activity in thalamus, insula and cingulate cortex
Orme-Johnson, *et al.*, 2006	fMRI, TM effects on brain reactivity to pain (N = 24)	Long-term TM practitioners 40-50% fewer voxels responding to pain in thalamus and total brain. Also in PFC and ACC

## Prefrontal Hypothesis and Transient Hypofrontality

The frontal lobe, though not involved in the generation of religious experiences like vision, voice and unification with the divine, is however known to affect personality and social function. The study on 12 healthy volunteers', six of whom religious and six non- religious, showed that religious experience may be a cognitive process, mediated by a pre-established neural circuit, involving dorsolateral, prefrontal, dorsomedial, frontal and medial parietal cortex. There is substantial evidence from the psychology of religion to suggest that people are prepared ‘for religious experiences and this readiness’ is probably mediated by the dorsomedial frontal cortex, leading to the commonly reported felt immediacy of religious experience (Azari *et al*., 2001). The experience, however, becomes religious when people consciously identify the experience as consistent with their own religious schema. This cognitive process most probably involves the dorsolateral, prefrontal and medial parietal cortex. Therefore, the prefrontal regions mediate both the preparedness of religious experience and conscious cognitive process involved in the appreciation of religious experience (Azari, *et al.*, 2001).

Imaging study of meditation reveals that the process of meditation which requires intense focus of attention, seems to activate the PFC (prefrontal cortex) (bilaterally but more on the right), as well as the cingulate gyrus. Thus, meditation appears to begin by activating the prefrontal and cingulate cortex associated with the will or intent to clear one's mind of thoughts or to focus on an object. Studies on the guided type of (externally guided word generation) meditation however show a decrease in frontal activity when compared to volitional (internal) word generation. Thus, prefrontal and cingulate activation may be associated with the volitional aspects of meditation (Newberg and Iversen, 2003).

Further, it is proposed that a balanced function of the medial PFC is needed to maintain balanced religious activities. The specific functions involved in this regulation of religion are those mediating compliance to rules and customs, self-reflection and the understanding of thoughts and feelings of others with compassion and empathy (theory of mind). Theory of mind (ToM) or mentalization is the ability to recognize that someone else has a mind different from one's own. It involves the ability to infer someone else's mind by facial expression, tone of voice and non-verbal communication. It involves the area concerned with action imitation, face imitation and intention understanding (IFG). The neural structures involved in ToM include IFG, STS and IPL on the right side, medial prefrontal cortex including the anterior cingulate cortex (ACC) orbitofrontal cortex (OFC), precuneus, somatosensory cortex, amygdala and the occipital cortex. Therefore the MNS is integral to the theory of mind (Gabbard, 2005; Siegal and Varley, 2002). The medial PFC is involved in error detection and monitoring and compliance to social norms and therefore is involved in mediating compliance to rules and customs. The medial PFC along with the posterior cingulate is involved in self-reflective thought and this helps the person to have an insight into his own experience and the perception of self in relation to the divine being. The third regulatory function, which includes the theory of mind, involves the medial PFC, especially the orbitofrontal cortex, the lesion of which impairs theory of mind tasks. Based on this it is hypothesized that the hypofunction of the medial PFC results in decreased religiosity (hyporeligiosity). This would result from a combination of reckless and lawless behaviour (impaired error detection), self-indulgence (loss of self reflection) and an inability to consider the thoughts of others (impaired theory of mind). Hyperfunction of the medial PFC, on the other hand, will lead to rigid conformity with rules and customs, excessive concern over oneself and one's existence and excessive interpretation of the mind of others, all of which results in heightened religiosity (hyperreligiosity). A balanced function of the medial PFC results in normal error detection, self-reflection and theory of mind resulting in a balanced religious activity (Muramoto, 2003).

It is also hypothesized that the mental states commonly referred to as altered states of consciousness seen during certain spiritual/religious practices are principally due to transient prefrontal cortex deregulation. Supportive evidence for this comes from psychological and neuroscientific studies of dreaming, endurance running, meditation, daydreaming, hypnosis and various drug-induced states. It is proposed that transient hypofrontality is the unifying feature of all these altered states and that the phenomenological uniqueness of each state is the result of the differential viability of various frontal circuits; and the hallmark of altered states of consciousness is the subtle modification of behavioural and cognitive functions that are typically ascribed to the prefrontal cortex (Dietrich, 2003). There is also evidence from a Positron Emission Tomography study of *Yoga Nidra* relaxation meditation, when compared with the normal resting conscious state, that meditation is accompanied by a relatively increased perfusion in the sensory imagery system: hippocampus and sensory and higher order association regions, with decreased perfusion in the executive system: dorsolateral prefrontal cortex, anterior cingulate gyrus, striatum, thalamus, pons and cerebellum (Lou, *et al.*, 2006).

So there is considerable evidence from imaging studies of many spiritual practices to suggest a role for the PFC in the mediation of spiritual and religious experiences.

## Thalamic Activation and PSPL Deafferentation

The PFC, when activated (functional imaging during meditative practices) via the glutamatergic projections, can activate the thalamus, especially the reticular nucleus of the thalamus, as part of a more global attentional network. The thalamus mediates the flow to the cortex of sensory information both visual and information needed to determine the body's spatial orientation via the lateral geniculate body (LGB) and the lateral posterior nucleus (LPN), respectively. The visual information is relayed via LGB to the striate (visual) cortex and the spatial information is relayed via LPN to the PSPL. When excited, the reticular nucleus via inhibitory GABAergic (gamma amino butyric acid) projections to the LGB and LPN cuts the input to the striate cortex and the PSPL (especially right). This functional deafferentation means a decrease in the arrival of distracting stimuli to the striate cortex and PSPL, enhancing the sense of focus during meditation.

**Figure 1:**
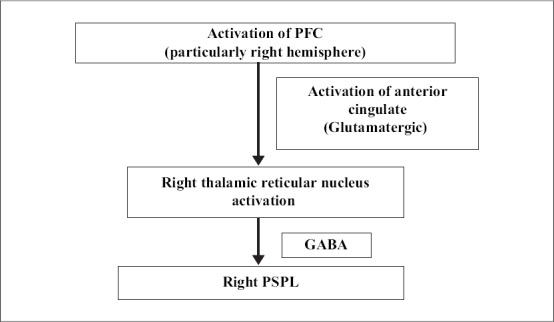
PSPL differentiation (Adapted from Newberg and Iversen, 2003)

The PSPL is involved in the analysis and integration of higher order visual, auditory and somaesthetic information and is also part of the complex attentional network including the PFC and thalamus. The PSPL helps construct a complex three-dimensional image of the body in space, helps distinguish objects and helps identify objects that can be grasped and manipulated. These functions help distinguish self and the external world and a deafferentation of this area is important in the physiology of meditation. This deafferentation results in an altered perception of self-experience during spiritual or meditative practices. The PSPL deafferentation is supported by three neuroimaging studies, all of which showed decreased activity in the region during intense meditation [Figure 1] (Newberg and Iversen, 2003).

## Activation of Hippocampus and Amygdala and the Hypothalamic and Autonomic Changes

**Figure 2:**
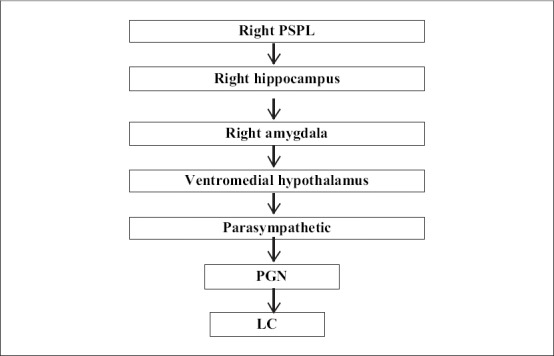
Neural circuitry of meditation post PSPL differentiation (Adapted from Newberg and Iversen, 2003)

Limbic stimulation is also implicated in experiences similar to meditation. The hippocampus modulates cortical arousal and responsiveness via its connections and hippocampal stimulation decreases cortical arousal and responsiveness. However, if cortical arousal itself is at a low level initially, then hippocampal stimulation augments cortical arousal. The partial deafferentation of the right PSPL during meditation results in the stimulation of the right hippocampus due to the inverse modulation of the hippocampus in relation to cortical activity. The right hippocampus influences the right lateral amygdala and they interact with each other in the generation of attention, emotion and certain types of imagery which are part of the experience of meditation. Functional imaging study (MRI) in Kundalini Yoga supports this notion of increased activity of hippocampus and amygdala in meditation (Newberg and Iversen, 2003). Stimulation of the right lateral amygdala results in stimulation of the ventromedial hypothalamus with stimulation of the peripheral parasympathetic system. The increased parasympathetic activity is associated with a subjective sensation, first of relaxation and later, a more profound sense of quiescence. Activation of parasympathetic system results in decreased heart and respiratory rate. All these physiological responses are observed during meditation. There is a decrease in innervation of the locus coeruleus (LC) by the paragigantocellular nucleus (PGN) when the heart rate and respiration slows down. This results in decreased noradrenaline, a finding seen in urine and plasma studies of subjects practicing meditation. The decrease PGN and LC stimulation cuts the supply from LC to the PSPL and LPN (via decreased noradrenaline) and decreases the sensory input on the PSPL contributing to the deafferentation. The LC also delivers less noradrenaline to the hypothalamic paraventricular nucleus (PVN) and decreases the production of corticotrophin releasing hormone (CRH) and cortisol. The urine and plasma studies show decreased cortisol level during meditation [Figure 2] (Newberg and Iversen, 2003).

## Sympathetic Activation: Breakthrough and Left PSPL Activation

There is evidence from a recent study for the mutual activation of parasympathetic and sympathetic axis in meditation (Newberg and Iversen, 2003). The evidence was based on the analysis of two meditative practices, which showed an increased variability of heart rate, suggesting the activation of both arms of the autonomic system. This also fits the descriptions of meditative states, which are associated with a sense of overwhelming calmness as well as significant alertness. The proposed mechanisms of the sympathetic activity include the breakthrough of sympathetic activity and the notion that some meditative practices activate the lateral hypothalamus via left hemispheric stimulation resulting in sympathetic drive.

**Figure 3:**
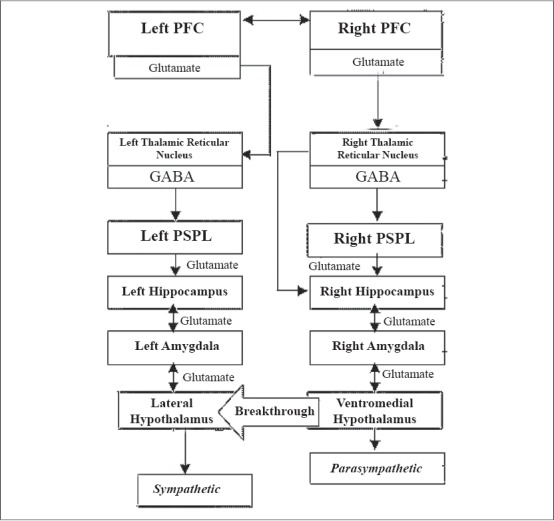
Sympathetic breakthrough (Adapted from Newberg and Iversen, 2003)

The intense stimulation of either the sympathetic or parasympathetic axis, if continued, could ultimately result in simultaneous discharge of both systems. This is considered as a ‘breakthrough’ of the other system. Meditative practices predominantly activate the parasympathetic system characterized by the low heart rate and respiratory rate associated with meditation. The continued parasympathetic stimulation ultimately results in a breakthrough of the other arm resulting in a sympathetic drive [Figure 3].

**Figure 4:**
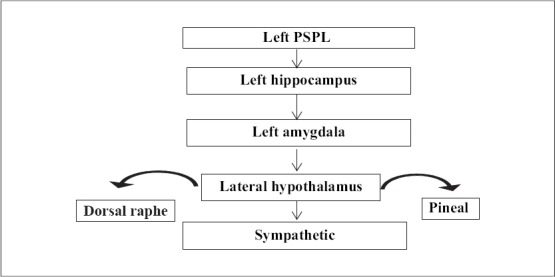
Left hemispheric neural sequence (Adapted from Newberg and Iversen, 2003)

There is lack of clarity as to the hemispheric stimulation that initiates the sequence of neural events during meditation. The model shows that the activity begins in the right hemisphere, but meditative practices might activate the left hemisphere first or cause bilateral activation. Further breakthrough might help with the stimulation of brain structures in both hemispheres. The left PFC activates the thalamus leading to a deafferentation of the left PSPL, which, via left hippocampus and amygdala, activates the lateral hypothalamus. The lateral hypothalamus in turn activates the sympathetic system; additionally it activates the serotoninergic dorsal raphae and the melatoninergic pineal gland [Figure 4].

## Temporal Lobe and Spirituality

Medical literature has all too frequently highlighted the temporal lobe as an area implicated in religious activity. The evidence for this is drawn from observations that temporal lobe epilepsy is characterized by religious experiences as part of the ictus and the inter ictal behaviour. Further, many psychophysiological ictal phenomena, like hallucinations, déjà vu, depersonalization etc are tagged to limbic system activation. These observations were analyzed and a limbic marker hypothesis of religion was proposed (Saver and Rabin, 1997). This states that limbic system tags ordinary experiences as profoundly important, as detached, as united into a whole and as joyous and such profound experiences could form the basis of religious experience. There are also suggestions that the superior temporal lobe may play a more important role than PSPL in body spatial representation (Karnath, *et al.*, 2001). However, this needs to be substantiated and the relationship between temporal and parietal lobe need to be further elucidated. In spite of the evidence pointing to a temporal lobe involvement, there is no literature, which shows that lesions or removal of temporal lobe resulted in a change in religious activity. It is therefore possible that temporal lobe, though associated with the psychophysiological phenomena interpreted with religious meaning, may not be necessary in maintaining religious activity (Newberg and Iversen, 2003; Muramoto, 2003).

## Neurochemistry of Spirituality

The dopaminergic system, via the basal ganglia, is involved in cortical subcortical interactions and a PET study on the dopaminergic tone in Yoga Nidra using 11 C-raclopride showed significant increase of dopamine during meditation (Kjaer, *et al.*, 2002). During meditation, 11C-raclopride binding in ventral striatum decreased by 7.9%. This corresponds to a 65% increase in endogenous dopamine release. It is hypothesized that the increase in dopamine is associated with the gating of cortical-subcortical interactions, leading to an overall decrease in readiness for action that is associated with this type of meditation. However more studies are needed to elucidate the role of dopamine and its interaction with other neurotransmitters in meditation (Kjaer, *et al.*, 2002).

There is also an increase in the serotonin levels during meditation, especially via the lateral hypothalamic stimulation of the dorsal raphae. This is correlated by findings of increased serotonin metabolites in the urine after meditation (Walton, *et al.*, 1995). Serotonin has effects on depression and anxiety and 5HT_2_ receptor stimulation can result in hallucinogenic effects. Serotonin, via the mechanism of inhibition of the LGB, greatly reduces the passage of visual information and this results in visual hallucinations, a phenomena seen when psychedelics with serotoninergic mechanism, like lysergic acid diethylamide (LSD) and psilocybin, are taken. This decrease in LGB action, along with increased reticular nucleus inhibition, increases the fluidity of temporal visual associations in the absence of sensory input, resulting in internally generated imagery described during certain meditative states. Serotonin increase can interact with dopamine and this link may enhance the feelings of euphoria seen during meditation. Serotonin, also in conjunction with glutamate, can result in the release of acetylcholine from Nucleus Basalis. While no studies have evaluated the role of acetylcholine in meditation, it appears that this neurotransmitter enhances attentional processing and spatial orientation during progressive deafferentation of input to PSPL (Yoshida, *et al.*, 1984; Newberg and Iversen, 2003).

Meditation associated with a decrease in the levels of noradrenaline, the mechanism of which as described earlier, is due to increased parasympathetic activity dampening PGN and resulting in decreased activity of the LC. The breakdown products of noradrenaline are generally found to be low in the urine and plasma during meditation (Walton, *et al.*, 1995).

There is an increase in the levels of the amino acid neurotransmitters, namely glutamate and GABA, during meditation. The increased PFC activity produces an increase in the level of free synaptic glutamate in the brain. Glutamate activates the N-methyl-D-aspartate (NMDA) receptors (NMDAr), but an excess of glutamate can kill these neurons through excitotoxicity. It is proposed that if glutamate levels reach excitotoxic levels during intense meditation, the brain might limit the production of N-acetylated-α-linked-acidic dipeptidase, the enzyme that converts the endogenous NMDAr antagonist N-acetylaspartylglutamate (NAAG) to glutamate (Thomas, *et al.*, 2000). NAAG is analogous to the dissociative hallucinogens like ketamine and phencyclidine and can produce such states. Therefore the NMDAr antagonist accumulation can produce a variety of states, like out of body and near death experiences, that are characterized as schizophrenomimetic or mystical (Newberg and Iversen, 2003). Reticular nucleus activation is the chief mechanism responsible for increase in GABA. Several studies have demonstrated an increase in serum GABA during meditation (Elias, *et al.*, 2000). GABA of course plays an important role in PSPL deafferentation.

Meditation is associated with a sharp increase of plasma melatonin (Tooley, *et al.*, 2000). Stimulation of the pineal gland by the lateral hypothalamus is responsible for the hike in melatonin. The increased melatonin may result in the calmness and decreased awareness of pain seen during meditation. It is also noted that during heightened activation, pineal enzymes synthesize 5-methoxy-dimethyltryptamine (DMT), which is a powerful hallucinogen. Several studies have linked DMT to out of body experience, time space distortion and other such mystical states (Strassan and Clifford, 1994).

Parasympathetic activation and decreased LC stimulation of the PVN of the hypothalamus as discussed above also results in decreased CRH and cortisol levels during meditation. The parasympathetic activation also results in decreased baroreceptor stimulation and secondarily releases its inhibition of the supraoptic nucleus, leading to the release of arginine vasopressin (AVP) and returns the blood pressure to normal. There is a dramatic AVP increase during meditation, which plays a role in decreasing self-perceived fatigue, increases arousal and helps consolidate new memories and learning. Increase in glutamate also stimulates the arcuate nucleus of the hypothalamus and causes the release of β-endorphin (BE). This is probably responsible for effects such as decreased pain and joyous and euphoric sensations during meditation along with other chemical mediators (Newberg and Iversen, 2003).

## Spirituality and Psychiatric Disorders: Neural Correlates of Anxiety

Meditation due to the neurochemical changes can produce an anxiolytic effect. The factors decreasing anxiety during meditation are an increased parasympathetic activity, decreased LC firing with decreased noradrenaline, increased GABAergic drive, increased serotonin and decreased levels of the stress hormone cortisol. The increased levels of endorphins and AVP also contribute to the anxiolytic effects of meditation (Newberg and Iversen, 2003).

## Depression

Spiritual practices can have considerable antidepressant effects due to the associated increase in serotonin and dopamine. Additional factors like increased levels of melatonin and AVP contribute to the antidepressant effects. There is an observed increase of β-endorphin as also NMDAr antagonism during meditation, both of which have antidepressant effects. The decreased level of CRH and cortisol also plays an important role in allaying depression. Thus, via multiple neurochemical changes, spiritual practices can counteract depression [Table 2 and 3] (Newberg and Iversen, 2003).

**Table 2:** Neurochemical Changes During Meditation (Adapted from Newberg and Iversen, 2003)

Neurochemical	Observed change

Dopamine	Increased
Serotonin	Increased
Melatonin	Increased
DMT	Increased
Noradrenaline	Decreased
Acetylcholine	Increased
Glutamate	Increased
NAAG	Increased
GABA	Increased
Cortisol and CRH	Decreased
AVP	Increased
β-endorphin	Increased

**Table 3:** Neurobiological Correlates of Anxiolytic, Antidepressant and Psychotic Affects During Spiritual Practice (Adapted from Newberg and Iversen, 2003)

Phenomenon	Neurobiological correlates

Anxiolytic effect	↓LC firing
Antidepressant effect	↓CRH and cortisol
Psychotic effect	↑Parasympathetic activity
	↑GABA
	↑Serotonin
	↑AVP
	↑β-endorphin
	↑Serotonin
	↑Dopamine
	↑β-endorphin
	↑Melatonin
	↑AVP
	NMDA antagonism
	↓CRH and cortisol
	5HT_2_ receptor activation
	↑Dopamine
	↑NAAG
	↑DMT

## Psychotic States

Meditation can induce psychotic states via mechanism such as increased 5HT_2_ receptor activation, increased DMT, increased NAAG and increased dopamine. The mechanisms include the 5HT inhibition of LGB, the hallucinogenic effects of DMT, the dissociative hallucinogenic effects of NAAG and the action of increased dopamine in the temporal lobe. A variety of schizophrenomimetic effects can be seen as a result of these complex neurochemical changes.

## Meditation and Neuroplasticity

A recent study using MRI was conducted to assess the cortical thickness in 20 participants with extensive Insight meditation experience involving focused attention to internal experiences. The participants were typical Western meditation practitioners who incorporated their meditation practices with their careers and family life. The study showed that brain regions associated with attention, interoception and sensory processing like the PFC and right anterior insula were thicker in meditation practitioners in comparison with matched controls. The prefrontal cortical thickness was most pronounced in older participants, suggesting that meditation probably offsets age-related cortical thinning. It was also noted that the thickness of PFC and right anterior insula correlated with meditation experience. The data provides structural evidence for experience-dependent cortical plasticity associated with meditation practice implying that meditation practices promote neuroplasticity (Lazar *et al*., 2005).

## Conclusion

Spiritual practices have shown definite neuroanatomical and neurochemical changes in the few studies that have been conducted so far to explore the neurobiology of such phenomenon. The evidence has been drawn mainly from studies that have examined meditation. However, they are replete with investigational constraints, methodological errors, small sample size and the results of many of the studies have not been replicated. There is need for further exploration of many of the prevalent spiritual/religious practices to clearly elucidate the neural correlates of the positive and negative effects they produce on physical and mental health.

### Take Home Message

Meditative practices have a positive impact on mental health. The neurobiological correlates during meditation partly explain the beneficial role. Meditative practices may augment psychotherapeutic interventions.

## Questions That This Paper Raises

1. What are the neural changes brought about by the different spiritual practices (meditation and others)?
2. Can all the beneficial effects of meditation be solely explained by the proposed neurochemical alterations?
3. What are the confounding variables that influence the neuroimaging study of meditation?
4. Is it possible to do real time neuroimaging studies on meditative/spiritual practices on a large sample of practitioners?
5. Is the right brain more important than left in spiritual practices?
6. What is the contribution of the left-brain as far as spiritual experience is concerned?

## About the Author

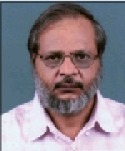



E. Mohandas, Postgraduate in psychiatry from the All India Institute of Medical Sciences, Delhi, India. Has served as consultant in Zambia. For the last 23 years, working as senior consultant at Elite Mission Hospital, Trichur, Kerala. Currently Chairman, Indian Association of Biological Psychiatry; CME director, South Asia Forum for Mental Health International; Award Committee Chairperson, Indian Psychiatric Society. Past President, Indian Association for Private Psychiatry.
